# Are the conventional scoring systems efficient in predicting mortality of acute mesenteric ischemia?: Mortality estimation in patients with AMI

**DOI:** 10.1097/MD.0000000000032619

**Published:** 2022-12-30

**Authors:** Arda Sakir Yilmaz, Necdet Fatih Yasar, Bartu Badak, Ahmet Murat Sendil, Mustafa Salis, Setenay Oner

**Affiliations:** a Departament of General Surgery, Sivrihisar State Hospital, Eskisehir, Turkey; b Department of General Surgery, Faculty of Medicine, Osmangazi University, Eskişehir, Turkey; c Cifteler State Hospital, Eskisehir, Turkey; d Department of Biostatistics, Faculty of Medicine, Eskisehir Osmangazi University, Eskisehir, Turkey.

**Keywords:** APACHE II, factor analysis, mesenteric ischemia, organ dysfunction scores, simplified acute physiology score, statistical analysis

## Abstract

Acute mesenteric ischemia is a surgical emergency with high morbidity and mortality rates. Therefore, it is important to determine the prognosis for this disease. In the present study, we aimed to compare the prediction accuracy of 3 scoring systems: Acute physiology and chronic health evaluation II, sequential organ failure assessment score and simplified acute physiology score II (SAPS II). The retrospective cohort study was conducted in a university hospital. Eighty-two acute mesenteric ischemia patients were evaluated retrospectively. The mortality prediction abilities of the scoring systems were evaluated by comparing the prediction rates of > 10%, 30% and 50% and the actual mortality among survivors and non-survivors in pairs. Predicted mortality rates among survivors and non-survivors differed among the 3 classification systems. The mortality estimates of the SAPS II were closer to the actual mortality rates. Analysis of the estimated mortality rates as mortality risk limits showed that acute physiology and chronic health evaluation II was superior to sequential organ failure assessment score and SAPS II in estimating mortality rates, whereas SAPS II was more successful in detecting survivors. The estimated mortality rates of the 3 rating systems, the estimated mortality rates were higher in the non-survivor group than in the survivor group. The accuracy of the SAPS II in determining prognosis was relatively better.

## 1. Introduction

Acute mesenteric ischemia (AMI) is characterized by decreased tissue perfusion resulting in tissue hypoxia, depletion of cellular energy stores, and accumulation of toxic metabolites, which lead to cell death. Mortality from AMI is associated with the extent of ischemic necrosis of the gut, which results in sepsis and multi-organ failure.^[1,2]^ Untreated or delayed in treatment results in a mortality rate of over 95% while surgical treatment decreases the mortality rate to approximately 70%.^[3]^ As the duration of symptoms prolongs, mortality increases from 12% to 98% after 48 hours.^[4]^ For this reason, early diagnosis and revealing the severity of AMI is essential.

Scoring systems put forth mortality risks in patients in intensive care units, which leads to better management of healthcare expenses. The worst physiological parameters within the first 24 hours of hospitalization were used for calculations of the acute physiology and chronic health evaluation II (APACHE II) and simplified acute physiology score II (SAPS II) scores, 2 of the most commonly used scoring systems.^[5,6]^ The sequential organ failure assessment (SOFA) aims to predict mortality according to organ failure.

Only a few studies have used the conventional scoring systems for AMI, and none have compared these scoring systems.

## 2. Objective

The objective of this study was to compare the predictive ability of APACHE II, SOFA, and SAPS II for mortality from AMI.^[7–9]^

## 3. Methods

The study protocol was approved by the local ethical committee (IRB:2021-09-22).

The medical records of 82 patients with AMI between January 2010 and January 2021 were retrospectively analyzed. A diagnosis of the intestinal ischemia was confirmed both surgically and pathologically.

The scores of APACHE II, SOFA, and SAPS II scores were calculated using the calculation tools via the links below, respectively: https://clincalc.com/Icu-Mortality/APACHEII.aspx, https://clincalc.com/IcuMortality/SOFA.aspx, https://clin-calc.com/IcuMortality/SAPSII.aspx. The worst clinical findings and laboratory values within the first 24 hours in the intensive care unit were used for the calculation.

### 3.1. Statistical analysis

SPSS version 25 software (SPSS Inc., Chicago, Illinois) was used for statistical analysis. Because the data values were within wide ranges, and the SOFA scoring system presents the predicted mortality rates in ranges, it was necessary to categorize the patients into 4 groups according to their predicted mortality rates as follows: group 1, < 10%; group 2, between 10% and 29%; group 3, between 30% and 49%; and group 4, ≥ 50%.

First, we assessed the predictivity of the 3 scoring systems for mortality by comparing estimated mortality rates across mortality groups (survivors/nonsurvivors) using the chi-square test. We then compared actual mortality in mortality groups with estimated mortality rates of > 10%, 30% and 50% for each scoring system system.

## 4. Results

The demographic data of patients, actual death rates, estimated mortality rates, and length of hospital stay are presented in Table 1. We confirmed the ability of the 3 scoring systems to predict death by showing that the estimated mortality rates differed between the surviving and non-surviving groups. The actual mortality rates in the estimated mortality groups are shown in Table 2, where the SOFA and SAPS II scoring systems were better able to estimate the actual mortality rates. In addition to predictive accuracy, we assessed the proportion of patients who died with > 10%, 30%, and 50% of the predicted mortality rates, and the proportion of patients who survived with mortality rates of < 10%, 30%, and 50% (Table 3 and Table 4, respectively). The mortality and survival predictions of the scoring systems were statistically compared in pairs (Table 5 and Table 6). Accordingly, it was observed that SOFA and SAPS II predicted higher mortality higher, and SAPS II was more successful in predicting survival.

**Table 1:** Estimated mortality rates calculated according to demographic data, length of stay, actual mortality rates and scoring systems of patients with AMI.

			n	%		*P*
Gender	Female		38	46.3		
n = 82	Male		44	53.7		
Mortality			38	46.3		
Comorbidities	Diabetes mellitus		51	62.2		
	Hypertension		41	50		
	Coronary artery disease		26	31.7		
	Atrial fibrillation		25	30.5		
	Chronic obstructive pulmonary disease		8	9.8		
	Cerebrovascular accident		14	17.1		
	History of thrombosis		10	12.2		
			Median	%25	%75	
Age			69	60	80	
Length of stay in the hospital			9	4	13	
APACHE II (all patients)	Severity score		17	11.75	22	
	Estimated mortality: %		42.1	28.07	60.1	
	Severity score	Survivors	13	9	16.5	*P* < .001
	Severity score	Nonsurvivors	21.5	18	26	
SOFA (all patients)	Severity score		4	2	9	
	Estimated mortality: %		<10	<10	15-20	
	Severity score	Survivors	3	2	4	*P* < .001
	Severity score	Nonsurvivors	8	5	12	
SAPS II (all patients)	Severity score		39.5	26.75	54.5	
	Estimated mortality: %		23.85	7.72	56.42	
	Severity score	Survivors	31.5	22.5	38.5	*P* < .001
	Severity score	Nonsurvivors	54	42	61	

AMI = acute mesenteric ischemia, APACHE II = acute physiology and chronic health, SAPS II = simplified acute physiology score, SOFA = sequential organ failure assessment.

**Table 2:** Actual mortality of the patients in the groups and their rates within the groups.

Estimated mortality groups		APACHE II	SOFA	SAPS II
Group 1	<10%	1/2 %50	14/52 %26.9	1/21 %4.7
Group 2	10–29%	13/27%48.1	6/11%54.5	9/27%33.3
Group 3	30–49%	13/21%61.9	12/13%92.3	6/10%60
Group 4	>50%	11/32%34.4	6/6%100	22/24%91.6

APACHE II = acute physiology and chronic health, SAPS II = simplified acute physiology score, SOFA = sequential organ failure assessment.

**Table 3:** Non-survivor rates relative to predicted death rates.

Predicted mortality rates	APACHE II	SOFA	SAPS II
≥10%	37/80	24/30	37/61
≥30%	24/53	18/19	28/34
≥50%	11/32	6/6	22/24

APACHE II = acute physiology and chronic health, SAPS II = simplified acute physiology score, SOFA = sequential organ failure assessment.

**Table 4:** Survivor rates relative to predicted death rates.

Predicted mortality rates	APACHE II	SOFA	SAPS II
<10%	1/2	38/52	20/21
<30%	15/29	43/63	38/48
<50%	23/50	44/76	42/58

APACHE II = acute physiology and chronic health, SAPS II = simplified acute physiology score, SOFA = sequential organ failure assessment.

**Table 5:** Comparison of mortality in the ≥ 10%, ≥30, ≥50 estimated mortality groups, respectively, among non-survivors.

		APACHE II	SOFA	SAPS II
APACHE II	≥10%		*P* < .01**	n.s.
	≥30%		*P* < .001***	*P* < .01**
	≥50%		*P* < .01**	*P* < .001***
SOFA	≥10%	**		n.s.
	≥30%	***		n.s.
	≥50%	*P* < .01**		n.s.
SAPS II	≥10%	n.s.	n.s.	
	≥30%	*P* < .01**	n.s.	
	≥50%	*P* < .001***	n.s.	

APACHE II = acute physiology and chronic health, n.s. = nonspecific, SAPS II = simplified acute physiology score, SOFA = sequential organ failure assessment.

*P < .05.

** *P* < .01.

*** *P* < .001.

**Table 6:** Comparisons of estimated mortality groups < 10%, <30%, and < 50% among survivors.

		APACHE II	SOFA	SAPS II
APACHE II	<10%	–	n.s.	n.s.
	<30%	–	n.s.	*P* < .05*
	<50%	–	n.s.	*P* < .01**
SOFA	<10%	n.s.	–	n.s.
	<30%	n.s.	–	n.s.
	<50%	n.s.	–	n.s.
SAPS2	<10%	n.s.	n.s.	
	<30%	*P* < .05*	n.s.	
	<50%	*P* < .01**	n.s.	

APACHE II = acute physiology and chronic health, SAPS II = simplified acute physiology score, SOFA = sequential organ failure assessment.

* *P* < .05.

** *P* < .01.

## 5. Discussion

The mortality rates of AMI are high if not treated early. When mesenteric blood flow is impaired, the intestines continue to be remain viable in the first 12 hours. However, if this period was between 12 to 24 hours, the preservation of intestinal viability decreased to 56%, and if it lasted longer than 24 hours, it decreased to approximately 18%. Therefore, early diagnosis and initiation of treatment as soon as possible will decrease the mortality rate.^[10,11]^ Most of the laboratory parameters for this purpose alone are not sufficient to determine the outcome in the early stage of AMI but are more useful in late-stage disease.^[12]^ For this reason, we evaluated the efficiency of the scoring systems to predict the prognosis of the disease in the early period.

In the present study, the mortality rate of patients with AMI was 46.3%, lower than that reported in the literature.^[13]^ We believe that the most important reason for this is that treatment could be started without delay after early diagnosis. The medical records revealed that the frequency of comorbid diseases, of which diabetes (62.2%) and hypertension (50%) were the most common, was higher than in previous studies.^[14,15]^ Regardless of the etiology, advanced age is an eminent risk factor for AMI.^[16]^ The mean age of the patients in our study was 69. Similarly, Hsu et al reported that the mean age of patients with mesenteric ischemia was 70 years.^[17]^ The median hospital stay of all patients and surviving patients was 9 days and 11 days, respectively. Caluwaerts et al^[18]^ reported a greater difference in the length of hospital stay between all patients and survivors. In their study, the average hospital stay of all patients with mesenteric ischemia was 13 days, while in the surviving patients, it was 33 days.^[18]^ We consider that this difference was due to the ratio of survivors to all patients.

Healthcare providers use mortality prediction models to manage resources and evaluate the quality of care and therapeutic interventions. In our study, we compared the APACHE II, SOFA, and SAPS II scoring systems, which are the most commonly used in our center.

In the present study, we found that the scores of the rating systems were higher in the non-survivor groups than in the surviving group, as shown in previous studies.^[7,8]^ The median APACHE II scores in both survival groups were higher than those reported by Wu et al^[7]^ (13 vs 11.9; 21.5 vs 14.3, respectively). On the other hand, the median SAPS II scores were lower than those reported by Piton et al^[8]^ have reported in the NUTRIREA2 trial (39 vs 59).^[8]^ Viswanathan et al^[19]^ have reported higher SOFA scores both in survivors and non-survivors compared to our study (3 vs 8.82; 8 vs 12.86, respectively).^[19]^

Even though the scores of all 3 systems were different in the survival groups, the predicted mortality rates using the SAPS II were more accurate than those using the APACHE II and SOFA scoring systems. Among patients with predicted mortality < 10%, SAPS II was the scoring system closer to the predicted mortality, whereas APACHE II and SOFA overestimated mortality. Within the 10% to 29% and 30% to 49% prediction widths, mortality estimates from all scoring systems were higher than the actual mortality rates. However, it was remarkable that the APACHE II scoring system underestimated mortality among patients with predicted mortality rates of > 50%.

When we analyzed the estimated mortality rates as mortality risk limits, we observed that even though APACHE II was superior to SOFA and SAPS II in estimating mortality among high-risk patients, APACHE II underestimated the mortality. Among the survivors, the SAPS II was the best system for predicting the survival rates.

Variations in the treatment strategies might have affected our results. In addition, while the SOFA scoring system aims more to detect organ dysfunction and morbidity, APACHE II and SAPS II are designed to detect mortality.^[20]^ Validation of our results in larger studies is requisite.

With these scoring systems, the subjective prognostic estimations and severity assessments can become more objective.

## 6. Conclusions

As a result, although all 3 scoring systems were predictive of the prognosis of AMI, they were far from perfect. The accuracy of SAPS II in determining prognosis was relatively better. This study indicates that it is necessary to develop a scoring system specific to mesenteric ischemia to improve mortality prediction.

## Author contributions

**Conceptualization:** Necdet Fatih Yasar.

**Data curation:** Ahmet Murat Sendil, Mustafa Salis.

**Formal analysis:** Setenay Oner.

**Investigation:** Ahmet Murat Sendil, Mustafa Salis.

**Methodology:** Arda Sakir Yilmaz, Necdet Fatih Yasar.

**Project administration:** Arda Sakir Yilmaz.

**Resources:** Arda Sakir Yilmaz, Mustafa Salis.

**Software:** Arda Sakir Yilmaz, Ahmet Murat Sendil.

**Supervision:** Bartu Badak.

**Validation:** Necdet Fatih Yasar, Ahmet Murat Sendil.

**Writing – original draft:** Arda Sakir Yilmaz.

**Writing – review & editing:** Bartu Badak.

## References

[1] Clair DG Beach JM. Mesenteric ischemia. N Engl J Med. 2016;374:959–68. 26962730 10.1056/NEJMra1503884

[2] Nuzzo A Maggiori L Ronot M. Predictive factors of intestinal necrosis in acute mesenteric ischemia: prospective study from an intestinal stroke center. Am J Gastroenterol. 2017;112:597–605. 28266590 10.1038/ajg.2017.38

[3] Copin P Zins M Nuzzo A. Acute mesenteric ischemia: a critical role for the radiologist. Diagn Interventional Imaging. 2018;99:123–34. 10.1016/j.diii.2018.01.004 29433829

[4] Ritz JP Germer CT Buhr HJ. Prognostic factors for mesenteric infarction: multivariate analysis of 187 patients with regard to patient age. Ann Vasc Surg. 2005;19:328–34. 15818455 10.1007/s10016-005-0005-5

[5] Knaus WA Draper EA Wagner DP. APACHE II: a severity of disease classification system. Crit Care Med. 1985;13:818–29. 3928249

[6] Le Gall JR Lemeshow S Saulnier F. A new simplified acute physiology score (SAPS II) based on a European/North American multicenter study. JAMA. 1993;270:2957–63. 8254858 10.1001/jama.270.24.2957

[7] Wu JM Tsai MS Lin MT. High APACHE II score and long length of bowel resection impair the outcomes in patients with necrotic bowel induced hepatic portal venous gas. BMC Gastroenterol. 2011;11:18. 21385464 10.1186/1471-230X-11-18 PMC3061950

[8] Piton G Le Gouge A Boisramé-Helms J. Factors associated with acute mesenteric ischemia among critically ill ventilated patients with shock: a post hoc analysis of the NUTRIREA2 trial. Intensive Care Med. 2022;48:458–66. 35190840 10.1007/s00134-022-06637-w

[9] Leone M Bechis C Baumstarck K. Outcome of acute mesenteric ischemia in the intensive care unit: a retrospective, multicenter study of 780 cases. Intensive Care Med. 2015;41:667–76. 25731634 10.1007/s00134-015-3690-8

[10] Filsoufi F Rahmanian PB Castillo JG. Predictors and outcome of gastrointestinal complications in patients undergoing cardiac surgery. Ann Surg. 2007;246:323–9. 17667513 10.1097/SLA.0b013e3180603010 PMC1933566

[11] Grootjans J Lenaerts K Buurman WA. Life and death at the mucosal-luminal interface: new perspectives on human intestinal ischemia-reperfusion. World J Gastroenterol. 2016;22:2760–70. 26973414 10.3748/wjg.v22.i9.2760 PMC4777998

[12] Lee MJ Sperry JL Rosengart MR. Evaluating for acute mesenteric ischemia in critically ill patients: diagnostic peritoneal lavage is associated with reduced operative intervention and mortality. J Trauma Acute Care Surg. 2014;77:441–7. 25159248 10.1097/TA.0000000000000381

[13] Schoots IG Koffeman GI Legemate DA. Systematic review of survival after acute mesenteric ischaemia according to disease aetiology. Br J Surg. 2004;91:17–27. 14716789 10.1002/bjs.4459

[14] Ozturk S Unver M Ozdemir M. Prognostic factors in acute mesenteric ischemia and evaluation with multiple logistic regression analysis effecting morbidity and mortality. Pol Przegl Chir. 2020;93:25–33. 33729172 10.5604/01.3001.0014.5824

[15] Wu W Yang L Zhou Z. Clinical features and factors affecting postoperative mortality for obstructive acute mesenteric ischemia in China: a hospital- based survey. Vasc Health Risk Manag. 2020;16:479–87. 33268989 10.2147/VHRM.S261167 PMC7701155

[16] Wadman M Syk I Elmståhl S. Survival after operations for ischaemic bowel disease. Eur J Surg. 2000;166:872–7. 11097154 10.1080/110241500447263

[17] Hsu HP Shan YS Hsieh YH. Impact of etiologic factors and APACHE II and POSSUM scores in management and clinical outcome of acute intestinal ischemic disorders after surgical treatment. World J Surg. 2006;30:2152–62. 17103101 10.1007/s00268-005-0716-3

[18] Caluwaerts M Castanares-Zapatero D Laterre PF. Prognostic factors of acute mesenteric ischemia in ICU patients. BMC Gastroenterol. 2019;19:80. 31146693 10.1186/s12876-019-0999-8 PMC6543602

[19] Viswanathan R Vivekanandan S Ravi A. Analysis of incidence and the value of SOFA and MOD scoring in predicting the outcome in acute mesenteric ischemia. Int Surg J. 2016;2:480–6.

[20] Xu F Li W Zhang C. Performance of sequential organ failure assessment and simplified acute physiology score II for post-cardiac surgery patients in intensive care unit. Front Cardiovasc Med. 2021;8:774935. 34938790 10.3389/fcvm.2021.774935 PMC8685393

